# Characteristics of central sensitization and its relationship with demographics and pain indicators among veterans with chronic pain: A cross-sectional study

**DOI:** 10.1097/MD.0000000000044054

**Published:** 2025-08-22

**Authors:** Aditi Tuell, Diane P. Dirette, Sarah L. Krein

**Affiliations:** a Interdisciplinary Health Sciences PhD Program, Western Michigan University, Kalamazoo, MI; b Dwyer School of Health Sciences, Indiana University South Bend, South Bend, IN; c Center for Clinical Management Research, VA Ann Arbor Healthcare System, Ann Arbor, MI; d Department of Internal Medicine, University of Michigan, Ann Arbor, MI.

**Keywords:** central sensitization, chronic pain, pain catastrophizing, pain interference

## Abstract

Central sensitization (CS) is a neural mechanism associated with the development and perpetuation of chronic pain. The purpose of this study was to examine the severity and prevalence of central sensitization and examine its relationships with demographics and pain indicators among veterans with chronic pain. This study used a cross-sectional design. Data for the Central Sensitization Inventory Part A (CSI-A), Pain Catastrophizing Scale, and Brief Pain Inventory-Short Form were manually extracted from paper forms and hospital electronic health records. Descriptive statistics, Kendall Tau correlation coefficient, and independent samples *t* test were used for data analysis. The sample consisted of 184 veterans, predominantly men (87%) with a mean age of 60 years. The mean CSI-A score was 45.06 ± 17.06 and 62.5% of veterans (n = 115) were identified as having CS (CSI-A ≥ 40), with 23.9% (n = 44) identified as having severe CS (CSI-A = 50–59). A significant but weak negative correlation was obtained between CSI-A score and age (τb = ‐0.121, *P* = .016) and a significant positive weak to moderate correlation was obtained between CSI-A score and severity of pain (τb = 0.209, *P* < .001), pain interference (τb = 0.333, *P* < .001), and pain catastrophizing (τb = 0.385, *P* < .001). Women veterans had a significantly higher mean CSI-A score compared to men (55.38 ± 13.23 vs 43.51 ± 17.06, *t*_df = 182_ = 3.260, *P* = .001). These findings highlight the high prevalence of CS among veterans with chronic pain, particularly in women, emphasizing the need to assess CS to improve chronic pain management in this population.

## 1. Introduction

The International Association for The Study of Pain defines chronic pain as, “pain that persists or recurs for longer than 3 months.”^[1p19]^ Data from the National Health Interview Survey indicates that in 2021, 20.9% of US adults and 32.0% of veterans experienced chronic pain.^[2]^ The higher prevalence of chronic pain among US veterans, generally defined as persons who served in the active military, naval, air or space service and were discharged under conditions other than dishonorable, is linked to frequent injuries sustained during deployments, the intense physical demands of military service, and a potential tendency to postpone seeking care for painful conditions.^[3,4]^ Joint and back pain are common among both men and women veterans with chronic pain, but compared to men, women veterans have higher rates of diagnoses indicating pelvic pain, fibromyalgia, headache, and systemic disorders causing pain.^[5]^ Veterans aged 50 to 64 years have the highest prevalence of chronic pain (30.8%) when compared to veterans aged 20 to 34 years (27.1%) and 35 to 49 years (27.7%).^[6]^

The development and perpetuation of chronic pain are influenced by a complex interplay of mechanisms within the central and peripheral nervous systems along with contributions from an array of psychosocial factors.^[7]^ Central sensitization (CS), along with pain catastrophizing, has been identified as a factor that influences the chronification of pain.^[7]^ CS is “an amplification of neural signaling within the central nervous system that elicits pain hypersensitivity.”^[8p55]^ CS involves structural, functional, and neurochemical changes in the central nervous system, resulting in 4 characteristic observable changes in individuals. These include: primary hyperalgesia, in which the individual becomes hypersensitive to a painful stimulus at the symptomatic areas, secondary hyperalgesia in which the individual becomes hypersensitive to a painful stimulus at a site remote from the symptomatic area, allodynia in which the individual becomes hypersensitive to nonpainful stimulus such as light touch, and global sensory hyperresponsiveness in which the individual becomes hypersensitive to a variety of stimuli such as bright lights, noise, medication, temperature, pressure, and chemicals.^[8]^

Despite the potential effect of CS in the development and treatment of chronic pain, only a few studies have examined the prevalence and severity of CS at the population level or among defined patient cohorts. A population-based survey by Haruyama et al^[9]^ found that central sensitization syndrome (Central Sensitization Inventory Part A [CSI-A] ≥ 40) was present in 4.2% of all survey participants and was significantly higher in women versus men (4.9% vs 2.7%, *P* < .001). Among patient groups with musculoskeletal pain the percentage with CS has ranged from 13% to 17% and appears to be more predominant among women compared to men.^[10–12]^ Studies examining CS severity have generally found the subclinical category as most prevalent. Ohashi et al,^[13]^ for example, identified subclinical CS (CSI-A ≤ 30) in 79% of patients with chronic hip pain, while Salaffi et al^[14]^ found 45.1% of patients with fibromyalgia reporting subclinical CS. Notably, these studies did not analyze CS severity separately for men and women. However, Abdon et al^[12]^ observed different patterns in patients with chronic neck pain, with 57.4% of women categorized as having moderate to extreme CS compared with 40.3% of men.

Studies have also indicated that CS is linked to pain severity, psychological factors, physical function, and age across various chronic pain conditions. Specifically, CSI-A scores have shown positive correlations with pain severity in people with knee osteoarthritis, chronic low back pain,^[15]^ fibromyalgia,^[16]^ and hip osteoarthritis.^[13]^ CSI-A scores are also positively correlated with depression and anxiety among people with knee osteoarthritis, chronic low back pain,^[15]^ chronic shoulder pain,^[17]^ and in women with fibromyalgia.^[16]^ Positive correlations between CSI-A scores and pain catastrophizing, a tendency to magnify the threat presented by a painful stimuli, have similarly been found among people with knee osteoarthritis, chronic low back pain,^[15]^ and chronic shoulder pain.^[17]^ Furthermore, CSI-A scores are negatively correlated with functional outcome measures, such as the 6-Minute Walk Test in patients with knee osteoarthritis, chronic low back pain,^[15]^ and arm endurance tests in patients with chronic shoulder pain.^[17]^ Finally, a negative correlation has been found between CSI-A scores and age in patients with hip osteoarthritis^[13]^ and in women with fibromyalgia.^[16]^

Although a higher proportion of veterans experience chronic pain, musculoskeletal injuries, polytrauma, and co-occurring mental health conditions,^[18,19]^ the prevalence, severity, and relationships of CS in this population has not been explored. Understanding the presence and potential impact of CS among US veterans is therefore a potentially important avenue for ensuring effective pain care delivery. The current study aimed to examine: the prevalence and severity of CS among veterans with chronic pain, the relationship of CS with age, the relationship of CS with severity of pain, pain interference, and pain catastrophizing, and the difference in reported CS between men and women veterans. Based on the literature, we hypothesized that: a quarter of the study population will present with CS and many will have moderate severity CS (CSI-A score 40–49); a positive linear correlation will exist between CS and severity of pain, pain interference, and pain catastrophizing, and a negative linear correlation will exist between CS and age; and women veterans will present with higher scores on the CSI-A when compared to men.

## 2. Materials and methods

### 2.1. Design

This study used a cross-sectional design involving manual data extraction, from paper forms and the electronic health record, of data collected as part of the chronic pain clinical program located at the Department of Veterans Affairs (VA) Community-Based Outpatient Clinic in Wyoming, MI (affiliated with the Battle Creek VA Medical Center, Battle Creek). This study was deemed exempt by the VA Ann Arbor Healthcare System and Western Michigan University Institutional Review Boards with a waiver of Health Insurance Portability and Accountability Act authorization and informed consent for all aspects of the project.

### 2.2. Study population

Veterans aged 18 years and older who completed the pain intake package following referral to the chronic pain clinical program between January 2021 and December 2023 were included in this study.

Using the population-based estimate of 32% for the prevalence of chronic pain in veterans,^[2]^ with an 80% power and 5% significance level 2-sided test (α = 0.05), a minimum sample size of 219 was determined for this study.

### 2.3. Data collection

Patients referred to the pain program are asked to complete a pain program intake package, which includes a personal health inventory, pain history questionnaire, and 3 established pain assessment instruments (the CSI-A, the Pain Catastrophizing Scale [PCS], and the Brief Pain Inventory-Short Form [BPI-SF]). This information is then entered into a note template in the electronic health record by pain program personnel. Data were manually extracted from the pain program intake package paper forms unless the package was not present in its entirety. In the latter case, data were manually abstracted from the electronic health record for patients who were identified by intake face sheets maintained by the pain program. The abstracted data were entered into an Excel file. Multiple measures were taken to ensure data accuracy with each entry checked 3 times and total scores for the PCS and CSI-A were also checked 3 times when the individual scores were available on the pain intake paper forms.

The final data file contained age (18–89 years with a separate categorical variable to indicate participant(s) ≥ 90 years), gender, self-reported pain location, severity, interference with walking ability, general activity, enjoyment of life, mood, work, relationships with other people, and sleep (from the BPI-SF), responses to the CSI-A and PCS.

### 2.4. Measures

The primary outcome measures for this study included the CSI-A, the PCS, and the BPI-SF. The CSI-A is a self-reported instrument that has been found to have psychometric strength, clinical utility, and construct validity in evaluating central sensitization-related symptoms in patients with chronic pain.^[20]^ The CSI-A uses a scale from 0 (never) to 4 (always) for 25 questions related to symptoms of CS. A cutoff score over 40 out of 100 is suggestive of CS.^[21]^ CSI-A scores can be divided into 5 categories based on severity, subclinical (0–29), mild (30–39), moderate (40–49), severe (50–59), and extreme (60–100).^[21]^

The PCS is a self-reported instrument used to evaluate catastrophic thinking related to pain in adult chronic pain patients. It is a 13-item scale, each question is answered using a 5-point Likert Scale of 0 (not at all) to 4 (all the time), scores range from 0 to 52, higher scores are indicative of higher levels of catastrophizing and a score >30 is indicative of clinically relevant levels of catastrophizing. PCS has good internal reliability (α = 0.92, 95% confidence interval 0.91–0.93) and test–retest reliability (Spearman ρ = 0.88, 95% confidence interval 0.83–0.93).^[22]^

The BPI-SF is a self-reported instrument used to identify the location of pain and measure the severity of pain and pain interferences. The instrument includes a body diagram where patients are asked to shade the areas where they feel pain and put an ``X’’ on the most painful area. The front and back of the body diagram are separated into 28 parts to report the total number of sites for pain. It also asks the patient to rate their worst, least, average, and current pain on a scale from 0 to 10 (0 = no pain and 10 = pain as bad as you can imagine). The recall period for the worst and least pain in the past 24 hours. The pain severity score is calculated as an average of the 4 items. The pain interference section consists of 7 items where patients are asked to describe pain interference with general activity, mood, walking ability, normal work, relations with other people, sleep, and enjoyment of life (0 = does not interfere and 10 completely interferes). The recall period for the pain interference section is the past 24 hours. The pain interference score is calculated as an average of the 7 items. This average can be used if at least 4 of the items have been completed by the patient.^[23]^ For the current study, the pain interference score was calculated as an average for interference with general activity, mood, walking, and enjoyment of life. The validity and reliability of the BPI was first established in patients with cancer pain but since has been demonstrated in multiple types of chronic non-cancer pain.^[24]^

### 2.5. Statistical analysis

The data were analyzed using IBM SPSS (Version 29.0.1.0; IBM Corp., Armonk). Descriptive statistics were used to examine the prevalence and severity of CS. The relationship between CSI-A score and age, the severity of pain, pain interference, and pain catastrophizing was determined using Kendall Tau correlation coefficient. The independent sample *t* test was used to examine the difference in the mean score for CSI-A between men and women veterans. The significance level for this study was set at *P* < .05 and all estimates are reported with 95% confidence interval when relevant. Missing data were analyzed using Little MCAR test, which indicated that the data were missing completely at random (*χ*^2^ (220) = 235.306, *P* = .228). For the CSI-A score, our primary variable of interest, 8.2% of the data were missing. For other variables, the percentage of missing data ranged from 2.7% for the total number of sites with pain to 26.6% for pain interference with walking ability. Missing data were replaced using the expected maximization method.^[25]^ The percentages for missing data for each variable are presented in File S1, Supplemental Digital Content, https://links.lww.com/MD/P744.

## 3. Results

The study sample consisted of 184 veterans, predominantly men (n = 160, 87%) with a mean age of 60 years. Patients in the study sample also had a mean pain duration of 19.38 years, reported multiple pain sites (mean 6.27) and a mean PCS of 29.5, with clinically significant levels of pain catastrophizing (PCS = 30–52) identified in 54.9% (n = 101). The demographic characteristics and pain-related measures are reported in Table 1.

**Table 1:** Demographic characteristics and pain-related measures (N = 184).

	Mean (SD)
Age (yr)	60.46 (13.70)
Duration of pain (yr)	19.26 (13.66)
Number of sites with pain	6.28 (4.59)
Severity of pain	6.24 (1.54)
Pain interference	7.03 (1.99)
PCS	29.51 (13.20)
CSI-A	45.06 (17.06)

CSI-A = Central Sensitization Inventory Part A, PCS = Pain Catastrophizing Scale.

The distribution of participants across the 5 severity categories of CS according to CSI-A scores is reported in Table 2. The mean CSI-A score was 45.01 ± 17.06 and 62.5% of veterans were identified as having CS (n = 115, CSI-A ≥ 40). Moreover, 23.9% were in the severe CS category (n = 44, CSI-A = 50–59) and 18.5% were in the extreme category (n = 34, CSI-A = 60–100).

**Table 2:** Distribution of participants across the 5 severity categories of central sensitization according to CSI-A scores.

	*n* (%)
Subclinical (0–29)	34 (18.5%)
Mild (30–39)	35 (19.0%)
Moderate (40–49)	37 (20.1%)
Severe (50–59)	44 (23.9%)
Extreme (60–100)	34 (18.5%)

CSI-A = Central Sensitization Inventory Part A.

The correlations between the CSI-A score and age, severity of pain, pain interference, and pain catastrophizing are presented in Table 3. A significant negative, weak correlation was identified between the CSI-A score and age (τb = ‐.121, 95% CI [‐.22, ‐.02], *P* = .016). A significant positive, but weak correlation was identified between the CSI-A score and severity of pain (τb = .209, 95% CI [.11, .31], *P* < .001), a significant positive, weak to moderate correlation was identified between the CSI-A score and pain interference (τb = .333, 95% CI [.24, .43], *P* < .001), and a significant positive, moderate correlation was identified between the CSI-A score and pain catastrophizing (τb = .385, 95% CI [.29, .48], *P* < .001).

**Table 3:** Correlations between the CSI-A score and age, severity of pain, pain interference, and pain catastrophizing.

	τb	*P*
Age	‐.121*	.016
Severity of pain	.209**	<.001
Pain interference	.333**	<.001
Pain catastrophizing	.385**	<.001

CSI-A = Central Sensitization Inventory Part A.

* Correlation is significant at .05 level (2-tailed).

** Correlation is significant at .01 level (2-tailed).

A significant difference was identified in the mean CSI-A score between men (43.51 ± 17.06, n = 160) and women veterans (55.38 ± 13.23, n* *= 24), *t*(182) = 3.260, 95% CI [4.68, 19.05], *P* = .001 with a large effect size (Cohen *d = *.714).

## 4. Discussion

CS is a neural mechanism associated with the development and perpetuation of chronic pain and appears to have a negative effect on many pain-related outcomes. This study found that 62.0% of veterans referred for chronic pain care were identified as having CS. This estimate is substantially higher than prior studies of patient populations with musculoskeletal pain, which found prevalence rates of <20%.^[10,11]^ The high prevalence of CS in this study population could be attributed in part to a higher incidence of musculoskeletal injuries and co-occurring mental health conditions among veterans.^[18,19]^ Nonetheless, further work to understand factors contributing to as well as the impact of CS among veterans with chronic pain is warranted.

This study also found a high severity of CS, with the highest percentage (23.9%) of veterans classified in the severe CS category. Several studies support the occurrence of CS in varying magnitudes in patients diagnosed with various diseases and pain conditions. For example, higher severity of CS has been found to a greater degree in patients diagnosed with fibromyalgia, traumatic neck pain, chronic fatigue syndrome, tension-type headache, migraine, temporomandibular disorder, and chronic pelvic pain.^[26]^ On the other hand, medium severity of CS has been found in some patients diagnosed with conditions such as chronic low back pain, nontraumatic neck pain, post-cancer pain, osteoarthritis, rheumatoid arthritis, upper extremity tendinopathies, and lower severity of CS in a small proportion of patients diagnosed with shoulder pain and lower limb tendinopathies.^[26]^ While the current study did not categorize veterans based on their specific type of pain, it identified that on average veterans reported pain in multiple body parts (6.27 ± 4.66) such as head, neck, shoulders, elbow, wrists, hands, upper, mid and lower back, abdomen, buttocks, hips, knees, ankles, and feet.

The analysis showed a significant negative but weak correlation between CSI-A score and age. This finding aligns with studies of patients with hip osteoarthritis and women with fibromyalgia, which also found a negative correlation between CS and age.^[13,16]^ The negative correlation may be attributed to the changes in the interpretation of pain and response style as people age. For example, it has been found that healthy community-dwelling older people are more reluctant to report pain as they may consider pain to be a normal part of aging.^[27]^ However, this finding is not consistent across people with different pain conditions who seek intervention in pain programs.^[27]^ The correlation between age and CS may be influenced by confounding variables such as duration of pain, medication use, and lifestyle. Additionally, the CSI may not adequately capture age-related changes in how the central nervous system processes pain, suggesting the need for further study.

A significant positive but weak to moderate correlation was also found between the CSI-A score and pain severity, interference, and catastrophizing. This finding corresponds with prior research, which found a positive correlation between CSI-A scores and severity of pain in people with hip osteoarthritis,^[13]^ knee osteoarthritis, chronic low back pain,^[15]^ and those with chronic shoulder pain.^[17]^ Similarly, previous studies suggest the CSI-A score is positively correlated with pain catastrophizing in people with knee osteoarthritis, chronic low back pain,^[15]^ and chronic shoulder pain.^[17]^ It has been found that CS causes an increase and persistence in pain sensitivity.^[8,26,28]^ Also, CS activates pain-enhancing pathways and impairs pain-inhibitory mechanisms.^[29]^ These changes at the level of the central nervous system may lead to greater interference with functional activities.

Previous studies have found a higher prevalence of CS in women.^[9,12]^ Likewise, we found a significant difference in the mean CSI-A scores between women and men veterans, with women veterans scoring higher. It has been found that women exhibit greater pain sensitivity. Also, differences in biological factors such as gonadal hormones, and endogenous pain modulation may contribute to sex differences. Differences in psychosocial mechanisms used by men and women may also help to explain the higher scores for CSI-A in women. It has been found that women report higher pain severity and catastrophizing then compared to men.^[30]^

## 5. Limitations

Data were obtained from a sample of veterans referred to a chronic pain program at a single site. In addition, some veterans did not complete the intake package, and some provided incomplete responses. As a result, the final study sample consisted of 184 patients, falling short of the number (n = 219) determined by our power calculation. These issues limit the potential generalizability of the study results and future work with a broader sample is needed. Second, this analysis focused on CS prevalence and the bivariate association between CS and a limited set of factors. Future studies that include a larger sample and use robust regression models are needed to determine what factors are predictive of CS for veterans with chronic pain and the effect of CS on key pain-related outcomes. Lastly, all outcome measures were patient-reported. Future studies should include objective measures such as conditioned pain modulation, pressure pain thresholds, and qualitative sensory testing to assess CS.

## 6. Implications for clinical practice and further research

Limitations notwithstanding, our findings showed a high prevalence and more severe CS among this sample of veterans with chronic pain, as well as correlations between CS, age, severity of pain, pain interference, and pain catastrophizing, which have important implications for their care. Screening veterans with chronic pain for CS is important for ensuring appropriate treatment. Effective management of CS often requires a multidisciplinary approach, including pain specialists, mental health professionals, and rehabilitation experts, along with medications that target the central nervous system. Given the high prevalence and severity of CS in this population, resources and research funding should be allocated to train healthcare professionals in recognizing and managing this condition. The significantly higher CSI-A scores for the subset of women veterans also suggest a need for heightened attention to this group in particular, and that future research investigating chronic pain in veterans should include gender as a key variable to better understand and address potential sex-related differences in pain experiences.

## 7. Conclusion

A high proportion of veterans were identified as having CS and approximately a quarter of these veterans were identified as having severe CS. CS had significant positive correlations with severity of pain, pain interference, and pain catastrophizing and significant negative correlations with age. Women veterans scored higher on the CSI-A when compared to men. The findings underscore the importance of examining CS in all veterans as this will facilitate the development and delivery of a comprehensive chronic pain management program.

## Acknowledgments

The authors would like to thank the staff and patients at the Wyoming VA Outpatient Clinic in Wyoming, MI, and Drs Christin Dejong and Adrianna Hooper for their invaluable support and guidance. The authors thank Dr Rob Lyerla, Interdisciplinary Health Sciences PhD Program, College of Health and Human Services, Western Michigan University, for his helpful review of statistical methods and analysis.

## Author contributions

**Conceptualization:** Aditi Tuell, Diane P. Dirette, Sarah L. Krein.

**Data curation:** Aditi Tuell.

**Formal analysis:** Aditi Tuell.

**Investigation:** Aditi Tuell.

**Methodology:** Aditi Tuell, Diane P. Dirette, Sarah L. Krein.

**Project administration:** Aditi Tuell.

**Resources:** Diane P. Dirette, Sarah L. Krein.

**Supervision:** Diane P. Dirette, Sarah L. Krein.

**Writing – original draft:** Aditi Tuell, Diane P. Dirette, Sarah L. Krein.

**Writing – review & editing:** Aditi Tuell, Diane P. Dirette, Sarah L. Krein.

## Supplementary Material


