# Supplementary material for: Characteristics of central sensitization and its relationship with demographics and pain indicators among veterans with chronic pain: A cross-sectional study
Source: Medicine (Baltimore). 2025 Aug 22;104(34):e44054. doi: 10.1097/MD.0000000000044054 (PMC12384800; doi:10.1097/MD.0000000000044054)
Supplement: Supplementary file 1 [file medi-104-e44054-s001.docx]

**Supplementary table**

Percentages of missing data for each variable

| Age | 0% |
| --- | --- |
| Gender | 0% |
| Duration of pain | 20.7% |
| Total number of sites with pain | 2.7% |
| Worst pain in the past 24 hours | 10.9% |
| Least pain in the past 24 hours | 10.3% |
| Average pain | 15.2% |
| Current pain | 23.4% |
| Pain interference with general activity | 13.0% |
| Pain interference with mood | 23.4% |
| Pain interference with walking ability | 26.6% |
| Pain interference with enjoyment in life | 10.3% |
| Pain Catastrophizing Scale Score | 15.8% |
| Central Sensitization Inventory Part A Score | 8.2% |
